# Metabolic Flexibility in Canine Mammary Tumors: Implications of the Carnitine System

**DOI:** 10.3390/ani11102969

**Published:** 2021-10-15

**Authors:** Nunzio Antonio Cacciola, Mariafrancesca Sgadari, Fabrizia Sepe, Orsolina Petillo, Sabrina Margarucci, Manuela Martano, Paola Maiolino, Brunella Restucci

**Affiliations:** 1 Department of Veterinary Medicine and Animal Production, University of Naples “Federico II”, Via F. Delpino, 1, 80138 Naples, Italy; mery.fra95@gmail.com (M.S.); fabriziasepe93@gmail.com (F.S.); manuela.martano@unina.it (M.M.); paola.maiolino@unina.it (P.M.); brunella.restucci@unina.it (B.R.); 2 Research Institute on Terrestrial Ecosystems (IRET), National Research Council (CNR), Via P. Castellino, 111, 80131 Naples, Italy; orsolina.petillo@cnr.it (O.P.); sabrina.margarucci@cnr.it (S.M.)

**Keywords:** canine mammary tumors, metabolic plasticity, carnitine system, β-oxidation

## Abstract

**Simple Summary:**

The process of cell transformation toward a malignant phenotype is generally due to genetic alterations and/or epigenetic changes, as well as rewiring of cellular signaling and reprogramming of metabolic pathways. In addition to glucose metabolism, cancer cells can derive fuel from fatty acid-β-oxidation (FAO), an important alternative bioenergetic pathway that is often dysregulated in cancer. Moreover, FAO enzymes (particularly components of the carnitine system) are overactivated in tumors, suggesting that they serve as metabolic signatures in various cancer cell types. Metabolic changes in carcinogenesis are a focus of current research and have been poorly studied in canine malignancies. We previously reported that CPT1A, the rate-limiting regulator of the FAO process, is deregulated in canine mammary tumor tissues and cells. In the present study, we examined the protein expression of the three remaining components of the carnitine system (CACT, CPT2, and CrAT) and confirmed their expression and deregulation in canine mammary tumor tissues and cells. We also found that low expression of carnitine system components was closely related to the malignancy grade of mammary tumors. Detailed studies to investigate the role of these components in canine mammary tumors are needed to also improve the therapeutic approach in dogs.

**Abstract:**

Deregulation of fatty acid catabolism provides an alternative energy source to glycolysis for cancer cell survival and proliferation. The regulator enzymes of the carnitine system (CS), responsible for the transport of fatty acids across mitochondrial membranes for β-oxidation are deregulated in tumorigenesis. Recently, we found that Carnitine Palmitoyl Transferase 1 (CPT1), a crucial regulator of CS components, is expressed and dysregulated in canine mammary tumor (CMT) tissues and cells. In this study, we examined the protein expression of the three remaining enzymes of CS (Carnitine Acylcarnitine Translocase (CACT), Carnitine Palmitoyl Transferase 2 (CPT2), Carnitine O-acetyltransferase (CrAT), in canine mammary cells and tissues by Western blot and immunohistochemistry. Protein expression of the components of CS was found in normal mammary glands and a concomitant deregulation of expression in CMT tissues that inversely correlated with the degree of tumor differentiation. Moreover, the expression and a different deregulation of CS-related proteins was also observed in CF33, CMT-U27, CMT-U309, and P114 cell lines used as in vitro model. These results demonstrate for the first time the expression of CS components in CMT tissues and cancer cells; however, further studies are needed to elucidate their roles in dogs as well.

## 1. Introduction

Cancer cells maintain their metabolic homeostasis under a variety of unfavorable conditions to which they adapt and in which they proliferate despite a hostile microenvironment [1]. Under such unfavorable conditions, cancer cells adjust their metabolism by activating alternative pathways to utilize different energy sources in the microenvironment [2]. One way to compensate for the high energy demand of malignant cell growth is glucose depletion [3]. Another way is to obtain energy by fatty acid oxidation (FAO) of lipids from adjacent adipose tissue, lipoproteins and phospholipids [4,5]. Thus, metabolic plasticity is an indispensable prerequisite for cancer cell growth and enables the activation of lipolytic and/or glycolytic metabolic pathways [6,7]. Fatty acid oxidation occurs in mitochondria and is catalyzed by the carnitine system (CS), which regulates the cytoplasmic transport of long-chain fatty acids [8]. Four components are involved in this transmembrane transport: (1) Carnitine Palmitoyl Transferase 1 (CPT1), which converts acyl-CoAs to acylcarnitine, (2) Carnitine Acylcarnitine Translocase (CACT), which catalyzes the exchange of acylcarnitine and carnitine between outer and inner mitochondrial membranes (3) Carnitine Palmitoyl Transferase 2 (CPT2), which converts acyl carnitine back to acyl CoAs for oxidation [9] and (4) Carnitine O-acetyltransferase (CrAT), which closes the carnitine cycle, catalyzes the addition or removal of carnitine from medium and short chain acyl-CoAs and regulates acetylcarnitine efflux from the mitochondrial matrix [10]. It has been suggested that these alterations and adaptations of cellular energy metabolism in neoplasms can be considered as “hallmarks” of malignancy [11]. Indeed, a link between mitochondrial dysfunction and malignancy has been established in several types of neoplasms, and CS appears to be an important player in malignant transformation [12,13,14,15]. Canine mammary tumors (CMTs) are the most commonly diagnosed neoplasms in intact females and not infrequently present a clinical challenge to veterinary oncology [16]. Several biomarkers for mammary gland neoplasms have been investigated to facilitate early tumor detection and have prognostic value [17]. In addition, CMTs represent a useful spontaneous animal model for human breast cancer because they share many biological, histological and molecular aspects [18,19,20]. In our recent study [21], we previously reported CS features of CMTs and investigated the expression of CPT1A, demonstrating deregulation of its expression in CMT cells and CMT tissues. Here, we used immunohistochemistry to examine the expression of the remaining three components of CS (CACT, CPT2, and CrAT) in a series of 32 spontaneous CMTs and compared their expression with the degree of tumor malignancy. The expression of CS proteins was also studied in CMT cells and tissues by western blot.

## 2. Materials and Methods

### 2.1. Cell Cultures

The cell lines used were described in our previous study [21]. CMT-U309 and CMT-U27 cells were cultured in Roswell Park Memorial Institute (RPMI) medium (Euroclone, Milan, Italy); P114 cells were maintained in Dulbecco’s Modified Eagle’s medium/Nutrient Mixture F-12 Ham (DMEM/F12) (Euroclone); and CF33 cells were cultured in Dulbecco’s modified Eagle’s medium (DMEM) (Euroclone). All cell lines were supplemented with 10% fetal bovine serum (Euroclone), 100 IU/mL penicillin/100 μg/mL streptomycin (Euroclone), and 2 mM L-glutamine (Euroclone), and grown in an atmosphere of 5% CO_2_ and 95% humidity at 37 °C. All cell lines were routinely tested for mycoplasma contamination.

### 2.2. Mammary Tissue Samples 

Mammary tissue samples were obtained from 32 bitches with malignant mammary tumors. The bitches underwent surgery at the Veterinary Teaching Hospital of the Department of Veterinary Medicine and Animal Production of Naples Federico II University. All specimens were from the pathology laboratory archives and had been previously used for diagnostic purposes. In addition, six macroscopically and histologically normal mammary tissue specimens were used as controls. These specimens were from cases in which the entire mammary gland chain had been removed according to the hospital’s surgical protocol. All samples were routinely divided into two aliquots and stored under appropriate conditions according to the diagnostic analyses to be performed. Histology and immunohistochemistry were performed on specimens fixed in 10% neutral buffered formalin and embedded in paraffin. For western blot analysis, samples were weighed, washed in phosphate buffer saline (PBS), and stored at −80 °C. Histologic diagnosis was performed on slides stained with hematoxylin and eosin (Supplementary Materials Figure S1) according to the updated classification and criteria of the Davis-Thompson DVM Foundation [22]. Histological grading of the tumor was performed according to the criteria proposed by Pena (2013) [23]: evaluation of tubule formation, nuclear pleomorphism and number of mitoses per 10 high power field (HPF). According to this criteria, malignant tumors were classified into three groups: 11 well differentiated (G1), 10 moderately differentiated (G2), and 11 poorly differentiated (G3).

### 2.3. Sample Preparation, Protein Extraction and Western Blot Analysis

Fresh tissue samples from normal canine mammary glands (NMGs) or mammary carcinoma tissues (CMTs) were cut into small pieces and homogenized in a lysis buffer containing: 25 mM Tris-Cl pH 7.4, 150 mM NaCl, 1% Nonidet P-40, 0.5% sodium deoxycholate, 0.1% sodium dodecyl sulfate, and 1× protease/phosphatase inhibitors (Roche, Basel, Switzerland), using an Ultra Turrax™ (Ika-Werke GmbH, Staufen, Germany). The tissue lysates were then sonicated three times for 15 s and centrifuged at 16,000× *g* for 20 min. Cell lysates were homogenized in the same lysis buffer, sonicated three times for 15 s and centrifuged at 16,000× *g* for 20 min. Protein concentration was determined using the BCA protein assay (Pierce, Rockford, IL, USA). Lysates were boiled for 5 min at 95 °C in β-mercaptoethanol-containing Laemmli Sample Buffer (Bio-Rad, Hercules, CA, USA) and separated by 8–10% SDS-PAGE before being electrotransferred onto nitrocellulose membranes (BioRad). Blot membranes were cut based on standard band positions and then incubated with the appropriate antibodies. Membranes were incubated overnight with the primary antibodies against SLC25A20 (CACT) (NBP1-86690, Novus Biological, Littleton, CO, USA), CPT2 (NBP1-85471, Novus Biologicals), CrAT (ab153699, Abcam). β-actin (sc-47778, Santa Cruz Biotechnology, Dallas, CA, USA) was incubated for 60 min at room temperature. Subsequently, the membranes were washed three times with the solution tris buffered saline containing 0.05% Tween^®^ 20. Immunoreactive bands were visualized by incubation with secondary horseradish peroxidase-conjugated antibodies for 60 min at RT using an enhanced chemiluminescence kit (Thermo Scientific, Rockford, IL, USA). Densitometry was performed using ImageJ software (National Institutes of Health, Bethesda, MD, USA).

### 2.4. Immunohistochemistry

Tissue sections were deparaffinized in xylene, dehydrated in graded alcohols, and washed in 0.01 M PBS pH 7.2–7.4. Endogenous peroxidase was blocked with 0.3% hydrogen peroxide in absolute methanol for 30 min. The immunohistochemical procedure (streptavidin-biotin peroxidase method LSAB kit; Dako, Glostrup, Denmark) has been described elsewhere [24]. Primary antibodies against SLC25A20 (CACT) (NBP1-76 86690, Novus Biological), CPT2 (NBP1-85471, Novus Biologicals), CrAT (ab153699, Abcam) were diluted 1:100 in antibody diluent (Dako) and applied overnight at 4 °C. The immunolabeling procedure included negative control sections incubated with normal serum IgG (Dako) in place of the primary antibody. A sample of canine duodenum was used as a positive control. A mixture of biotinylated anti-mouse and anti-rabbit immunoglobulins (LSAB kit; Dako) in PBS was used as secondary antibody and applied for 30 min. After washing in PBS, the sections were incubated with streptavidin conjugated to horseradish peroxidase in Tris-Cl buffer containing 0.015% sodium azide (LSAB kit; Dako) for 30 min. For the detection of immunolabeling, diaminobenzidine tetrahydrochloride was used as the chromogen and hematoxylin as counterstain. 

#### Scoring of Immunoreactivity

Immunoreactivity was assessed by two pathologists (BR and MS) in a blinded semiquantitative manner, taking into account, first, the number of positive cells in 10 high-power fields (samples were divided into 4 grades: grade 0: no positive cells, grade 1: <10%; grade 2: 10–30%; grade 3: 31–60%; grade 4: >60%) and, second, the intensity of staining, which was classified as weak (1), moderate (2), or strong (3). Then, a combined immunoreactivity score (IRs) between 0 and 12 was calculated for each sample by multiplying the values of these two classifications according to Burrai and colleagues [25].

### 2.5. Statistical Analysis

All data are expressed as mean ± standard error of the mean (S.E.M.). Comparisons were made using the Student’s *t*-test or one-way analysis of variance followed by Tukey’s post hoc test where appropriate. Statistical analyses were performed using GraphPad Prism v7.0 software (La Jolla, CA, USA), and differences were considered statistically significant when *p* < 0.05.

## 3. Results

We examined the expression profile of the components of CS in seven CMT tissues compared with three NMGs by western blot (WB) to assess antibody cross-reactivity and target specificity. 

**Figure 1:**
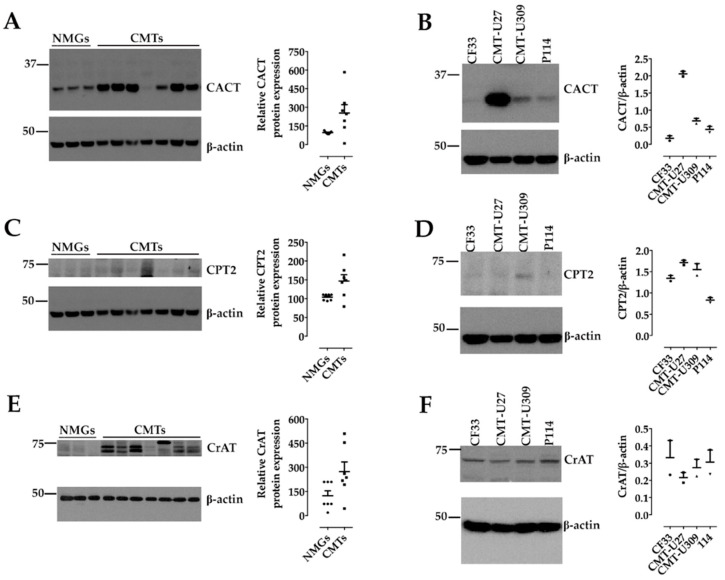
Expression of CACT, CPT2 and CrAT in Normal Mammary Gland (NMG) tissues, Canine Mammary Tumor (CMT) tissues and CMT cells: (**A**,**C**,**E**) Western blot analysis of CACT, CPT2 and CrAT protein expression in three NMG tissues and seven CMT tissues. β-Actin was used as a control to check the same protein loading. The graph shows the mean ± SEM of relative CACT, CPT2, and CrAT protein expression in CMT compared with NMG. (**B**,**D**,**F**): Representative Western blot showing CACT, CPT2, and CrAT protein expression in four CMT cells of different tumor origin (CF33: mammary adenocarcinoma; CMT-U27: simple ductal carcinoma; CMT-U309: spindle cell carcinoma; and P114: anaplastic carcinoma). Plots show densitometric analysis of CACT, CPT2 or CrAT protein expression expressed as CACT, CPT2 or CrAT/β-actin densitometry ratio for each cell line. β-Actin was used as a loading control to verify equal protein loading. Note that β-actin was identical for CACT and CPT2 in tissues and cell lines. See Materials and Methods and Supplementary Materials Figure S2.

Immunoreactive CACT bands with the predicted molecular weight and different signal intensity were detected in both normal and mammary tumor tissues (Figure 1A). However, densitometric analysis of the bands revealed that CACT protein expression was higher in CMT tissues than in NMG tissues, regardless of tumor malignancy grade (Figure 1A). CACT protein expression levels also differed when endogenous CACT levels were examined by WB on CMT cells. CMT-U27 expressed abundant CACT, whereas CF33, CMT-U309, and P114 expressed low levels of this protein (Figure 1B). 

Immunoreactive CPT2 bands were detected in both normal and mammary tumor tissues with the expected molecular weight and varying signal intensity (Figure 1C). Densitometric analysis revealed that the CPT2 protein was slightly more highly expressed in CMT tissue extracts than in NMG tissue (Figure 1C). In lysates of CMT cells, CPT2 expression was detected in all four malignant cell lines, with the lowest expression observed in P114 cells (Figure 1D). 

In NMG and CMT tissues, immunoreactive CrAT bands with the expected molecular weight and different signal intensities were observed (Figure 1E). Densitometric analysis revealed an increase in CrAT in CMTs compared to NMGs (Figure 1E). Similarly, WB analysis performed using whole cell lysates revealed bands of similar intensity in all four CMT cell lines used (Figure 1F).

In light of our findings on canine mammary tissues and cancer cells, we also investigated the protein expression of the components of CS in a series of CMT tissues and NMG tissues by immunohistochemistry (IHC). The results of IHC are summarized in Table 1. Notably, CACT, CPT2 and CrAT proteins were expressed only by ductal and lobular epithelial cells in both NMG and CMT. Myoepithelial cells were consistently negative.

**Figure 2:**
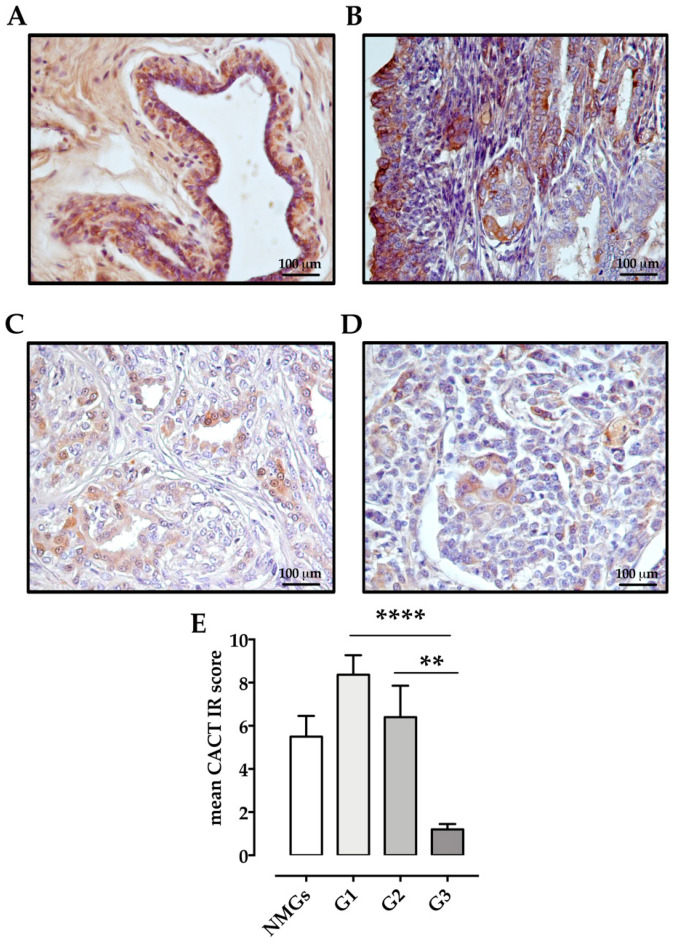
Immunohistochemical labeling of CACT in normal and neoplastic canine mammary glands: (**A**) Normal mammary gland (NMG), case number 4, IRs = 6: moderate CACT immunoreactivity, characterized by few and small cytoplasmic granules, is detectable in 31.3% of epithelial cells. (**B**) Well differentiated (G1) tubulopapillary carcinoma, case number 11, IRs = 12: strong CACT immunostaining is through the cell cytoplasm in 87.1% of neoplastic epithelial cells. (**C**) Moderately differentiated (G2) tubulopapillary carcinoma, case number 23, IRs = 1: weak CACT immunostaining is detectable in the cell cytoplasm of 3.7% of neoplastic epithelial cells. (**D**) Poorly differentiated (G3) tubular carcinoma, case number 30, IRs = 1: weak CACT immunoreactivity diffused through the cell cytoplasm is detectable in 1.4% of neoplastic epithelial cells. (**E**) Graph shows the mean ± SEM of immunoreactive (IR) score for the expression of CACT in normal and tumoral samples with different malignancy degree. **** *p* < 0.0001 G1 vs. G3 carcinomas; ** *p* < 0.01 G2 vs. G3 carcinomas. NMGs, normal mammary glands; G1, grade 1 carcinomas; G2, grade 2 carcinomas; G3, grade 3 carcinomas.

CACT expression was found in all tissue types: NMGs (*n* = 6/6 100%; mean IRs = 5.5 ± 0.957, range 1–8), G1 carcinomas (*n* = 11/11 100%; mean IRs = 8 ± 0.907, range 4–12), G2 carcinomas (10/10 100%; mean IRs = 6 ± 1.454, range 1–12) and G3 carcinomas (*n* = 9/11 82%; mean IRs = 1.6 ± 0.491, range 0–6). In NMGs, CACT immunoreactivity was moderate and characterized by few and small cytoplasmic granules (Figure 2A), whereas in CMTs 88% of G1 carcinomas had strong CACT immunostaining diffusing through the cell cytoplasm (Figure 2B), while CACT immunoreactivity decreased from G1 to G3 and from G2 to G3 carcinomas (Figure 2C–E).

**Figure 3:**
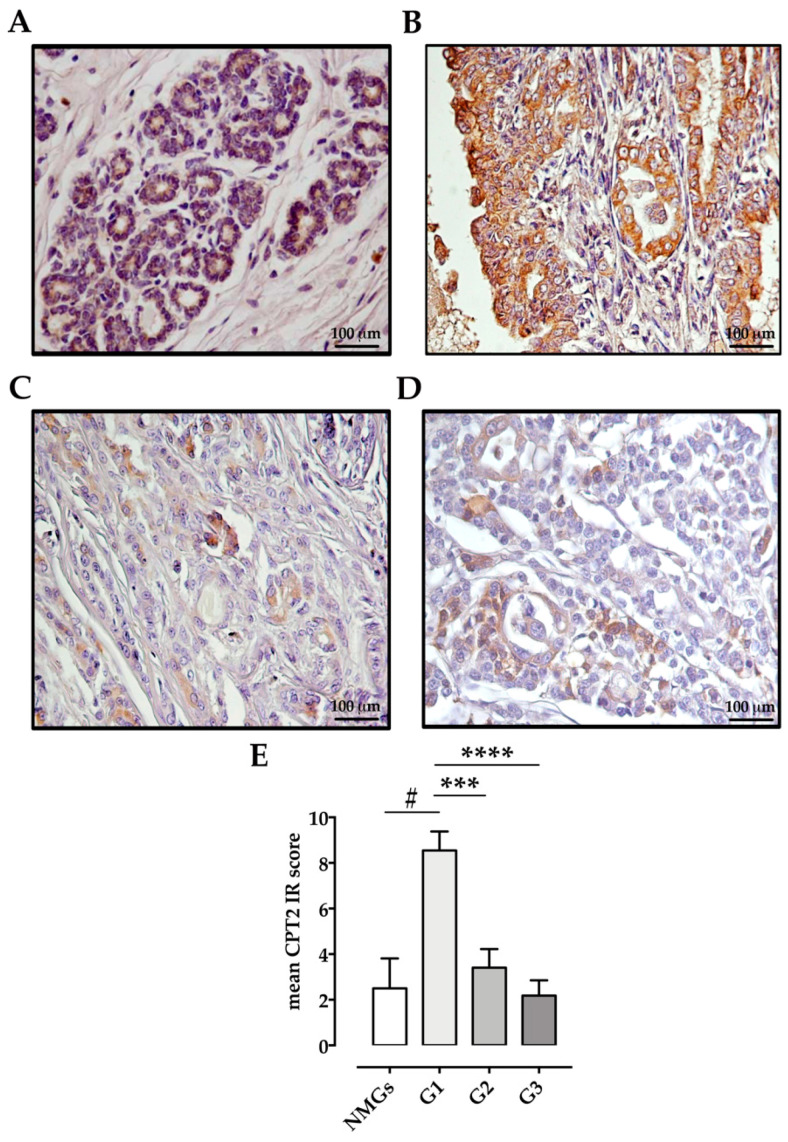
Immunohistochemical labeling of CPT2 in normal and neoplastic canine mammary glands: (**A**) Normal mammary gland (NMG). Case number 6, IRs = 1: Weak CPT2 immunostaining is expressed by 9% of ductal epithelial cells. (**B**) Well differentiated (G1) tubulopapillary carcinoma, Case number 11, IRs = 12: Strong immunostaining diffuses through the cell cytoplasm in 67% of neoplastic epithelial cells. (**C**) Moderately differentiated (G2) tubulopapillary carcinoma, case number 23, IRs = 1: weak CPT2 immunostaining is seen in the cell cytoplasm of 3.2% of neoplastic epithelial cells. (**D**) Poorly differentiated (G3) complex carcinoma, case number 28, IRs = 6 moderate immunostaining is expressed by 32.8% of neoplastic cells. (**E**) Graph shows the mean ± SEM of immunoreactive (IR) score for the expression of CACT in normal and tumoral samples with different malignancy degree. # *p* < 0.001 G1 vs. NMGs; *** *p* < 0.001 G1 vs. G2 carcinomas; **** *p* < 0.0001 G1 vs. G3 carcinomas. NMGs, normal mammary glands; G1, grade 1 carcinomas; G2, grade 2 carcinomas; G3, grade 3 carcinomas.

CPT2 expression was observed in all mammary tissues: in NMGs (*n* = 6/6 100%; mean IRs = 2.5 ± 1.31, range 1–9), G1 carcinomas (*n* = 11/11 100%; mean IRs = 8.55 ± 0.835, range 4–12), G2 carcinomas (10/10 100%; mean IRs = 3.4 ± 0.819, range 1–6), and G3 carcinomas (*n* = 8/11 73%; mean IRs = 2.18 ± 0.671, range 0–6). CPT2 immunoreactivity was weak in NMGs (Figure 3A) and increased in 72% of G1 carcinomas, with strong and diffuse cytoplasmic CPT2 immunostaining (Figure 3B). In G2- and G3-carcinomas, CPT2 protein expression was restricted to regions where mammary gland morphology was still preserved (Figure 3C,D). G2 and G3 tumors had lower mean CPT2 IRs compared with G1 tumors (Figure 3E).

**Figure 4:**
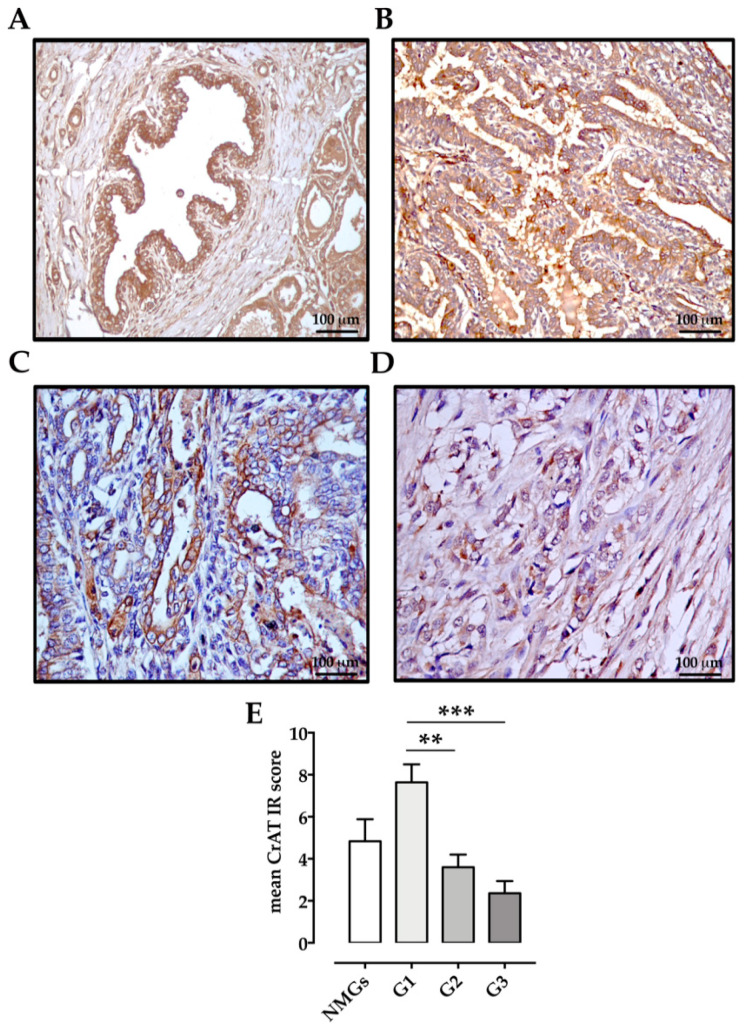
Immunohistochemical labeling of CrAT in normal and neoplastic canine mammary glands: (**A**) Normal Mammary Gland (NMG), case number 4, IRs = 8: moderate CrAT immunostaining is seen in 64.8% of ductal epithelial cells. (**B**) Well differentiated (G1) Tubular carcinoma, case number 16, IRs = 9: strong CrAT immunostaining, characterized by small granules, is seen in 66.5% of neoplastic epithelial cells. (**C**) Moderately differentiated (G2) tubular papillary carcinoma, case number 24, IRs = 4: moderate cytoplasmic CrAT immunoreactivity is expressed by 23.4% of neoplastic epithelial cells. (**D**) Poorly differentiated (G3) tubular carcinoma, case number 32, IRs =2: moderate immunostaining is evident in neoplastic epithelial cells infiltrating the surrounding tissue. (**E**) Graph shows the mean ± SEM of immunoreactive (IR) score for the expression of CrAT in normal and tumoral samples with different malignancy degree. ** *p* < 0.01 G1 vs. G2 carcinomas; *** *p* < 0.001 G1 vs. G3 carcinomas. NMGs, normal mammary glands; G1, grade 1 carcinomas; G2, grade 2 carcinomas; G3, grade 3 carcinomas.

CrAT expression was detected in all tissue samples, in NMGs (n = 6/6 100%; mean IRs = 4.83 ± 1.05, range 2–8), in G1 carcinomas (*n* = 11/11 100%; mean IRs = 7.64 ± 0.856, range 2–12), in G2 carcinomas (10/10 100%; mean IRs = 3.6 ± 0.6, range 1–6) and in G3 carcinomas (*n* = 9/11 82%; mean IRs = 2.36 ± 0.576, range 0–6). In NMGs, immunoreactivity was moderate and characterized by few and small cytoplasmic granules (Figure 4A). In contrast, 88% of G1 carcinomas showed strong and diffuse cytoplasmic immunoreactivity (Figure 4B). Moderate CrAT intensity was observed in G2 (Figure 4C) and G3 carcinomas (Figure 4D), especially in neoplastic cells infiltrating the surrounding tissue (Figure 4D). The mean CrAT IRs score was higher in G1-, than the G2 and G3-carcinomas (Figure 4E).

## 4. Discussion

In the tumor microenvironment, cancer cells reprogram their metabolic patterns to stimulate cell growth and proliferation [1]. Moreover, it has been suggested that the fatty acid oxidation (FAO) pathway may provide an alternative energy source for anabolic processes in various tumors; therefore, altering these pathways may be a promising target for therapy [26,27,28]. Our results show for the first time the expression of the components of CS (CACT, CPT2, and CrAT) in canine mammary tissues and cell lines, although the number of samples analyzed may seem relatively small in view of the high prevalence of mammary carcinomas in dogs; this may be a limitation of the study. The catabolic process by which fatty acids are broken down for energy is β-oxidation. Increased FAO rates havebeen found in several cancers, including lung, breast, liver [29] and prostate [30], suggesting that the carnitine shuttle system has an interesting potential that should be further investigated for diagnostic purposes. In addition, several studies have recently shown that receptor-positive human breast and prostate cancer cells (MCF-7 and C4-2B cells) exhibit a faster FAO rate compared to receptor-negative cells (MDA-MB-231 and PC-3 cells) [30,31].

To date, little attention has been paid to the relationship between CACT protein and cancer development, and only a few studies have described an association between altered expression of CACT and tumorigenesis [32,33]. We demonstrated that CACT was expressed in CMT samples and cancer cells. Moreover, by IHC, the strongest CACT expression was found in G1 carcinomas. CACT expression was negatively correlated with tumor malignancy, with CACT expression downregulated in poorly differentiated G3 carcinomas compared to NMGs, well-differentiated (G1) and moderately differentiated (G2) carcinomas. These results are consistent with a previous study in which the authors found decreased expression of CACT in hepatocellular carcinoma (HCC) cells and a significant correlation with poor survival in patients with HCC [32]. Consistent with our findings that CACT expression was downregulated in CMTs, another previous study confirmed that in human bladder cancer patients, the expression of CACT was significantly deregulated in cancer tissues compared with healthy bladder tissues [33]. 

Like CACT, CPT2 was also expressed in both CMT tissues and CMT cells. Similarly, using IHC, we found that CPT2 expression was increased in G1 carcinomas. Moreover, CPT2 protein expression correlated with the decrease in tumor differentiation. Little is known about the deregulation of CPT2 in cancer; however, a recent study reported that this enzyme can be considered as an independent prognostic factor in colorectal cancer patients [34]. Our results regarding CPT2 expression, which follows a similar pattern to CACT, are also consistent with previous findings showing that CPT2 is downregulated in HCC and is associated with tumor differentiation grade and vascular invasion [35]. Our results are also consistent with those of Zhang X. and coworkers (2021), who demonstrated downregulation of CPT2 in ovarian cancer (OC) cells and a poorer prognosis for patients with OC [36]. 

We also found altered CrAT expression in CMTs and CMT cells. That is, similar to the expression patterns of CACT and CPT2, CrAT protein levels also correlated negatively with an increase in tumor malignancy and had the lowest levels in the most malignant cell types (G3), again consistent with a previous report in which CrAT expression was significantly lower in human muscle-invasive bladder cancer compared with normal bladder tissue [33]. 

Thus, components of CS, including CPT1A, appear to be upregulated in low-grade mammary carcinomas and progressively downregulated or less expressed as the tumor becomes more malignant. Since neoplastic cell survival depends on the dynamics of nutrients present in the microenvironment [37], the adipose-rich mammary gland may represent a source of fatty acids required for an energy metabolism alternative to glycolysis. In this context, the components of CS could play an essential role, especially in well-differentiated (G1) tumors, to generate energy for their rapid growth and proliferation. In contrast, the loss and/or downregulation of CS components observed in moderately (G2) and less (G3) differentiated tumors could be due to the unfavorable and hypoxic tumor microenvironment, which primarily relies on the glycolytic metabolic pathway [38,39]. Thus, the importance of the components of CS in mediating the metabolic flexibility of CMTs may allow cancer cells to constantly adapt to changing intracellular and extracellular metabolic conditions.

## 5. Conclusions

In summary, the results of our study suggest that, as in humans, the components of CS are also expressed in CMT cells and deregulated in CMT tissues, confirming the role of the dog as an animal model for spontaneous neoplastic disease. Although mastectomy is still considered the most effective treatment, the existence and deregulation of these components in dogs may also provide new druggable targets for the prevention and treatment of canine mammary cancer. To this end, further studies are needed to also clarify their role also in dogs.

## Figures and Tables

**Table 1:** Main characteristics of normal and neoplastic canine mammary gland tissue and immunostained (IS) CACT, CPT2, and CrAT cells, staining intensity and immunoreactive (IR) score ^1^.

Normal Mammary Glands												
Case Number	Breed	Age (Years)	Histotype	CACT			CPT2			CrAT		
				IS Cells (%)	Staining Intensity	IR Score	IS Cells (%)	Staining Intensity	IR Score	IS Cells (%)	Staining Intensity	IR Score
1	Epagneul Breton	10		5	1	1	4	1	1	11	2	4
2	Mixed Breed	13		74.2	2	8	3	1	1	38.3	1	3
3	Yorkshire Terrier	8		38	2	6	12	1	2	18.4	2	4
4	Mixed Breed	10		31.3	2	6	36	3	9	64.8	2	8
5	Mixed Breed	9		49.4	2	6	9	1	1	11.9	1	2
6	Cocker	7		59.4	2	6	9	1	1	61.9	2	8
Well Differentiated G1 Carcinomas												
1	Mixed breed	14	Tubular carcinoma	31.4	3	9	49	3	9	45.9	3	9
2	Mixed breed	11	Tubular carcinoma	37.9	3	9	18.2	3	6	13.9	3	6
3	Yorkshire terrier	13	Complex carcinoma	11.9	2	4	17.6	3	6	48.7	3	9
4	Yorkshire terrier	8	Complex carcinoma	38.1	2	6	24.5	2	4	22.7	2	4
5	Mixed breed	7	Tubulopapillary carcinoma	87.1	3	12	67	3	12	84.5	3	12
6	Maltese	7	Mixed carcinoma	13.4	2	4	38.6	3	9	29.4	1	2
7	Mixed breed	13	Tubular carcinoma	24	3	6	60	2	8	38.7	3	9
8	Cocker	10	Tubular carcinoma	56.4	3	9	54.6	3	9	55	3	9
9	Cocker	9	Tubulopapillary carcinomay	74	3	12	75.2	3	12	72	2	6
10	Mixed breed	9	Tubular carcinoma	70.1	3	12	53.9	3	9	66.5	3	9
11	Beagle	6	Tubular carcinoma	57.1	3	9	78.6	3	12	65.3	3	9
Moderately differentiated G2 carcinomas												
12	Shih tzu	11	Tubular carcinoma	6.2	1	1	2.4	1	1	59	2	6
13	Epagneul breton	10	Tubulopapillary carcinoma	39	1	3	2.8	1	1	53.8	2	6
14	Cocker spaniel	10	Tubular carcinoma	58.3	3	9	64.1	2	8	24.7	2	4
15	Mixed breed	9	Solid carcinoma	78.4	3	12	1.3	1	1	9.3	2	2
16	Mixed breed	13	Tubular carcinoma	97.3	3	12	23	2	4	12.7	1	2
17	Mixed breed	9	Tubulopapillary carcinoma	3.7	1	1	3.2	1	1	1.2	1	1
18	Mixed breed	10	Tubulopapillary carcinoma	13.5	3	6	33.9	2	6	23.4	2	4
19	German sheperd	8	Tubulopapillary carcinoma	77	3	12	40.5	2	6	59.4	2	6
20	Mixed breed	8	Tubulopapillary carcinoma	14	1	2	26.2	1	2	33.4	1	3
21	Mixed breed	7	Mixed carcinoma	35.2	2	6	83	1	4	24.7	1	2
Poorly differentiated G3 carcinomas												
22	Greyhound	8	Tubular carcinoma	19.3	2	2	19.2	1	2	52.3	2	6
23	Mixed breed	10	Tubular carcinoma	3	1	1	2.5	1	1	4.5	1	1
24	Shih tzu	9	Tubular carcinoma	1.4	1	1	3.3	1	1	1.5	1	1
25	Greyhound	9	Tubular carcinoma	19.3	2	2	19.6	1	2	19.3	2	4
26	Mixed breed	9	Solid carcinoma	0.6	1	1	0	0	0	24.3	2	4
27	Mixed breed	13	Complex carcinoma	0	0	0	0	0	0	14.2	1	2
28	Mixed breed	13	Mixed carcinoma	1.4	2	2	32.8	2	6	0	0	0
29	Mixed breed	6	Complex carcinoma	4	2	2	53.3	2	6	12.5	1	2
30	Mixed breed	8	Tubular carcinoma	11.2	3	6	28.3	2	4	10.2	2	4
31	Poodle	15	Complex carcinoma	0	0	0	0	0	0	0	0	0
32	Mixed breed	9	Tubular carcinoma	3	1	1	25.9	1	2	10.3	2	2

^1^ The immunoreactive (IR) score ranges from 0 to 12 and is obtained by multiplying the percentage of immunostained CACT, CPT2, and CrAT cells (score: 0–4) by the staining intensity score (0–3). See Section 2 for more details.

## Data Availability

The data presented in this study are available on request from the corresponding author.

## Supplementary Materials

The following are available online at https://www.mdpi.com/article/10.3390/ani11102969/s1, Figure S1: Hematoxylin and eosin staining of histological types; Figure S2: whole western blots of Figure 1.
