# Supplementary material for: Metabolic Flexibility in Canine Mammary Tumors: Implications of the Carnitine System
Source: Animals (Basel). 2021 Oct 15;11(10):2969. doi: 10.3390/ani11102969 (PMC8532965; doi:10.3390/ani11102969)

Full original blots used for Figure 1 A, C, and E. Each blot membrane was cut based on the standard band positions and then incubated with the appropriate antibodies.

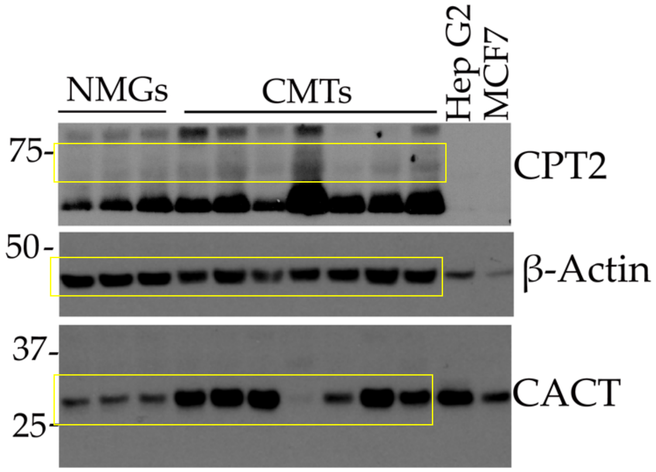


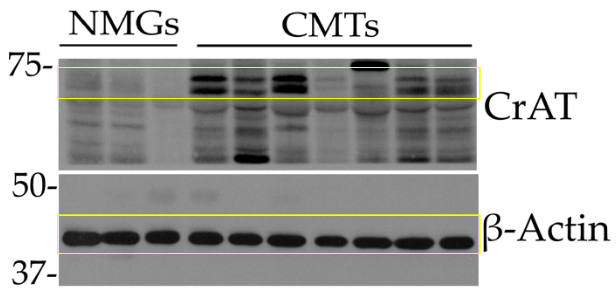


Full original blots used for Figure 1 B, D, and F. Each blot membrane was cut based on the standard band positions and then incubated with the appropriate antibodies.

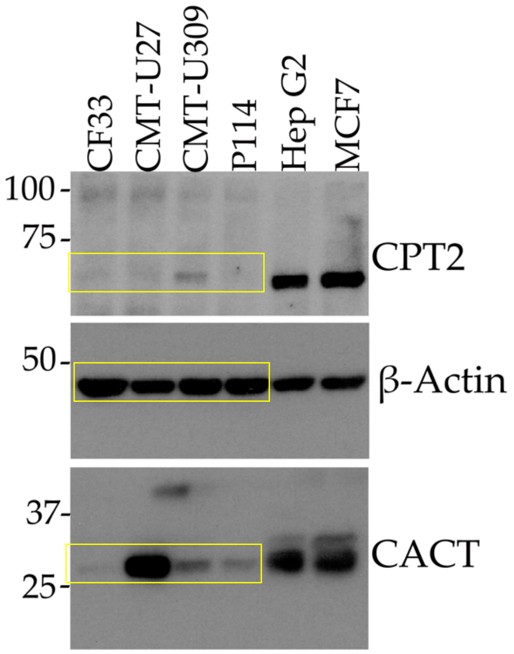


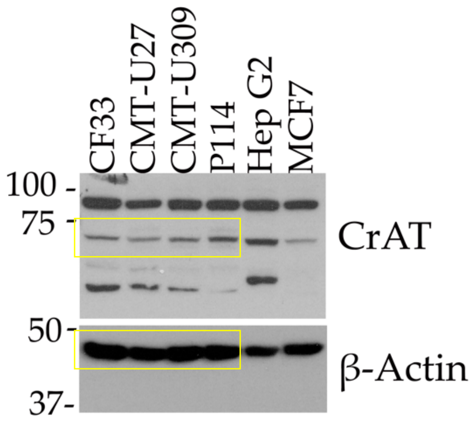

Supplement: Supplementary file 1 [file animals-11-02969-s001.zip › animals-1386933-supplementary.pdf]
